# From Sensation to Action: Neuroplasticity, Cognitive–Motor Training, and Emerging Biomarkers of Adaptation

**DOI:** 10.3390/brainsci16070749

**Published:** 2026-07-15

**Authors:** Carter Witbeck, Tony Montina, Gerlinde A. S. Metz

**Affiliations:** 1 Canadian Centre for Behavioural Neuroscience, Department of Neuroscience, University of Lethbridge, Lethbridge, AB T1K 3M4, Canada; 2 Department of Chemistry and Biochemistry, University of Lethbridge, Lethbridge, AB T1K 3M4, Canada; 3 Southern Alberta Genome Sciences Centre, University of Lethbridge, Lethbridge, AB T1K 3M4, Canada

**Keywords:** sensorimotor integration, cognitive–motor learning, neuroplasticity, virtual reality (VR), cognitive enhancement, rehabilitation, metabolomics, ^1^H NMR spectroscopy, metabolic biomarkers

## Abstract

Human interaction with the environment depends on the integration of sensory input, cognitive processing, and motor output within dynamic sensorimotor loops. These processes are supported by distributed neural circuits and shaped by learning, memory, and neuroplasticity across the lifespan. This review synthesizes current understanding of the mechanisms underlying sensorimotor integration and highlights how experience-dependent plasticity supports functional recovery and performance optimization in both health and disease. Disruptions such as traumatic brain injury, neurodegenerative disease, and aging may frequently result in combined cognitive and motor impairments. Here, we review non-invasive interventions that leverage neuroplasticity, including physical activity, motor training, and cognitive training, with increasing emphasis on integrated cognitive–motor approaches. Emerging technologies such as virtual reality provide ecologically valid, immersive environments that simultaneously engage perception, cognition, and action, with the potential to enhance training outcomes. However, variability in effectiveness and limited evidence for far transfer remain key challenges. To address these limitations, we highlight the integration of immersive training with objective biological measures. In particular, proton nuclear magnetic resonance (^1^H NMR)-based metabolomics offers a promising, non-invasive approach to identify biomarkers of neuroplastic adaptation. The integration of robust biomarker tools may facilitate the development and assessment of effective precision cognitive–motor interventions to optimize rehabilitation approaches and help build resilience in vulnerable individuals.

## 1. Introduction

The interaction between an organism and its environment is mediated by highly complex and dynamic biological processes. At any given moment, multiple sensory modalities, including vision, taste, smell, touch, and audition, enable the brain to continuously detect and process vast amounts of information. Much of this processing occurs outside of conscious awareness, allowing the brain to construct a coherent and integrated representation of the external world. Selective attention enables prioritization of behaviorally relevant stimuli, supporting goal-directed behavior, learning, and memory formation. The ability to acquire sensory information, integrate it with prior experience and context, and translate it into coordinated motor actions is fundamental to everyday functioning. These operations depend on the tightly coordinated activity of distributed neural circuits and interacting biological systems that typically function seamlessly. Disruption through injury, disease, or environmental challenge often reveals the interdependence and vulnerability of these systems. The present narrative state-of-the-art review examines how sensory, cognitive, and motor domains interact, outlines the underlying neuroanatomical substrates and circuits, and discusses the roles of learning, memory, and neuroplasticity in both recovery and performance optimization. It then considers current approaches to cognitive and motor training, with emphasis on methodological advances, ongoing debates, and emerging strategies aimed at improving functional outcomes. Although sensorimotor integration, neuroplasticity cognitive–motor training, virtual reality (VR), and metabolomics have each been largely studied independently, limited work has considered how these domains may be integrated within a single translational framework. This integration is important because cognitive–motor interventions are typically evaluated using behavioral outcomes alone, which may not fully capture the biological processes that contribute to individual differences in training response, retention, and transfer. Therefore, this review aims to combine foundational mechanisms with applied intervention strategies and emerging ^1^H NMR-based metabolomic approaches to support a translational perspective on training-related adaptation. This conceptual framework aims to inform the development of evidence-based cognitive and motor interventions with relevance for clinical, educational, and community settings.

### 1.1. Navigating Within Complex Environments: The Sensorimotor Loop

**Figure 1:**
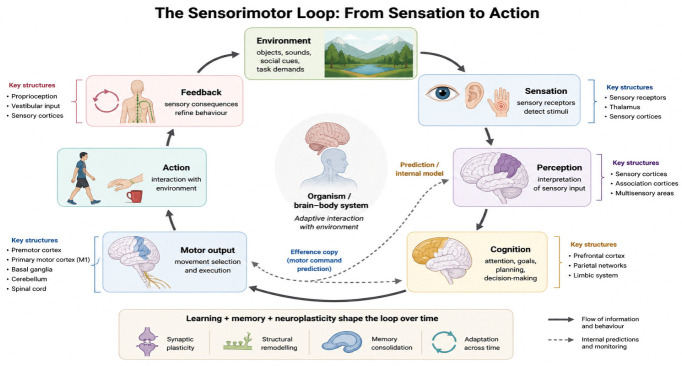
The Sensorimotor Loop: Integration of Sensation, Cognition, and Action. The sensorimotor loop links environmental stimuli to adaptive behavior through continuous interactions among sensation, perception, cognition, motor output, action, and feedback. Sensory feedback and internal predictions refine ongoing behavior, while learning, memory, and neuroplasticity modify the loop over time. This framework illustrates how cognition and motor function are dynamically integrated as a foundation for cognitive–motor learning in real-world scenarios and clinical training interventions.

To better understand the nature of the relationship between humans and their environment, it is essential to recognize that both are physically independent systems that interact through sensorimotor loops. The organism, consisting of a body and brain, cannot directly perceive the environment [1]. Instead, sensory organs are required to gather external stimuli and translate them into neural signals that the brain can process. This transformation enables the organism to construct a meaningful internal representation of its surroundings, thereby facilitating interaction and navigation. As illustrated in Figure 1, this cyclical exchange between sensory input and motor output is commonly referred to as the sensorimotor loop [2].

#### 1.1.1. Domains of the Sensorimotor Loop

The sensorimotor loop involves five domains of brain function: sensation, perception, cognition, motor, and feedback [1]. Sensation refers to the initial detection of stimuli from the external environment by sensory receptors, while perception involves the brain’s interpretation of these sensory inputs, resulting in conscious awareness of the environment [3]. Cognition is the capacity to realize a goal by organizing perceptual information, understanding rules, applying problem-solving strategies, and deciding the best plan of action [4]. The execution of the motor plan relies on the motor system with the goal of orienting and moving the body into a position that will produce the highest probability of achieving the goal and receiving a reward [5]. Finally, afferent sensory input acquired during ongoing motor actions plays a critical feedback role in refining movements toward achieving specific goals [6].

To conceptualize the sensorimotor loop, one can consider the cognitive–motor demands involved in playing a structured word-based board game, such as *Scrabble*. Initially, the player is presented with a set of scrambled letter tiles. The process of visually detecting these tiles represents sensory input, while recognizing and organizing them into potential words reflects perceptual processing. Subsequent cognitive operations involve strategic evaluation: determining the optimal word to play based on knowledge of game rules, current board configuration, and anticipated opponent responses. This stage engages executive functions such as planning, decision-making, and working memory. Once a decision is made, the motor system is engaged to execute the selected action, such as reaching, grasping, and placing the tiles on the board in a coordinated manner. Throughout gameplay, ongoing feedback from the sensory environment and opponent responses informs adjustments in strategy and motor behavior, illustrating the dynamic and recursive nature of sensorimotor integration. Ultimately, the word-based board game analogy provides a simple yet effective illustration of the sensorimotor loop, which helps explain how the human brain interacts with the environment in many everyday situations.

#### 1.1.2. Neurophysiology and Neuroanatomy of the Sensorimotor Loop

Understanding the neuroanatomical underpinnings of the sensorimotor loop remains a complex and ongoing challenge in neuroscience. A common example used to describe the sensorimotor loop relates to eye movements (saccades) toward a novel stimulus from a fixed point [7]. In this example, photons emitted from the newly presented stimulus reach the retina and interact with its photoreceptors (rods and cones). Electromagnetic radiation (light) is converted into electrical signals in photoreceptors through a biochemical cascade that alters membrane potential, leading to changes in neurotransmitter release at the photoreceptors’ synaptic terminals [8]. Exposure to light causes photoreceptors to hyperpolarize and reduce the amount of glutamate that is released. The reduction of glutamate influences the bipolar cells differently depending on whether they are ON- or OFF-type bipolar cells [8]. Nevertheless, the process leads to retinal ganglion cells sending electrical signals through the optic nerve, passing through the optic chiasm and continuing through the optic tract towards the lateral geniculate nucleus (LGN) of the thalamus, and relayed to the primary visual cortex (V1) [9].

Information about the form, color and motion begins to be processed and assembled as it makes its way through several visual regions of the occipital lobe [10]. This newly processed information is passed through the dorsal and ventral streams towards the parietal and frontal lobes, further to process the location and identity of the stimulus [11]. The increasingly processed information continues towards the frontal eye field (FEF) of the prefrontal cortex before continuing to the superior colliculus (SC) region of the midbrain [10]. Both the FEF and SC regions contain topographical ocular motor maps; therefore, visual afferent signals and efferent eye motor commands activate FEF and SC neurons [10,12]. The efferent signal sent by the SC and other brainstem regions commands the eye muscles to contract, reorienting the fovea of the eye towards the newly presented stimulus, while signals from the SC feed back to the cortex about the time, direction, and size of the saccade [10]. Although the visuomotor circuitry underlying saccades provides insight into the neuroanatomical nature of sensorimotor loops, it underrepresents the multifaceted nature of complex motor behaviors.

Several other nervous system regions play a role in complex movements, including the prefrontal cortex (PFC), premotor cortex, primary motor cortex (M1), basal ganglia, cerebellum and the spinal cord. When reaching for an apple, the PFC and premotor cortex work in tandem. While the PFC is assisting with decisions of which action to perform by integrating intention, motivation, rules and context, the premotor cortex is selecting and sequencing appropriate motor plans, combining both the contextual information provided by the PFC with external sensory information [13]. Once a decision has been made and a motor plan has been selected, the motor cortex executes the voluntary movement through the spinal cord. The spinal cord acts as a conduit between the motor cortex and the skeletal muscles, relaying signals to control the arm and hand [14]. In turn, the arm and hand work in unison to reach and grasp the apple and move it towards the mouth. Simultaneously, the basal ganglia and the cerebellum ensure that the desired movements are initiated and are conducted in a smooth and controlled manner, relying on both internal and external feedback [15]. Furthermore, both structures play an essential role in motor learning [16,17]. Together, the neurophysiology, neuroanatomy, and neurocircuitry discussed in this section provide a small window into the hardware underlying the human’s capacity to interact with their environment. When living within an ever-changing environment, however, key features of the sensorimotor loop are capable of learning and memory, allowing for the refinement and recall of skills.

## 2. Learning and Memory

While the sensorimotor loop enables real-time interaction with the environment, the brain’s ability to adapt and refine these interactions over time depends on learning and memory. These cognitive and motor processes allow us to store past experiences, predict future outcomes, and modify behavior to optimize performance in a dynamic world.

The acquisition of new skills and knowledge is considered learning. For learning to occur, practice of the desired outcome and forms of feedback to correct the behaviors are required. In turn, learning leads to changes in behavior that are retained over time. According to Fitts and Posner’s [18] model, motor skill learning progresses through three phases: cognitive, associative, and autonomous. During the cognitive phase of learning a motor skill, individuals attempt to understand the skill by discovering strategies and rules that will aid in their learning. Once the most effective strategies are known, a gradual emergence from the cognitive phase to the associative phase, and continuous practice, allows the learner to refine their skills. Over time, consolidation of the skill will occur, and little cognitive resources are necessary to perform the motor skill. At this stage, the individual is considered to be in the autonomous phase of learning, where performance is refined and becomes smooth and efficient. Improvements in speed and accuracy throughout these phases serve as behavioral indicators that learning is taking place [19].

An important part of learning is not just gaining new skills or knowledge but also retaining them for future use. Within Fitts and Posner’s [18] model, repeated practice during the three phases of learning a skill not only allows for the refinement of the skill, but each trial also leads to encoding of the skill into long-term memory, also known as consolidation [20]. Long-term memory can be separated into two categories: implicit memory and explicit memory [21]. Implicit and explicit memories are defined by the mode of memory retrieval [22]. Implicit memory is unconscious and is expected to result from the autonomous phase of learning [23]. For example, someone who has learned a motor skill performs the skill in response to an associated external cue, as the information is processed in a bottom-up manner. In contrast, explicit memory is conscious and intentional. For instance, actively recalling the experience of learning the motor task would be considered an explicit memory. Other examples of explicit memory relate to conscious recall of learned facts, such as remembering the capital of Canada or recalling a past vacation. Both implicit and explicit memories play important roles in daily life, supporting the formation of routines, the recall of information, and the ability to make adjustments. Together, implicit and explicit memory systems rely on neuroplastic changes that occur during learning, making neuroplasticity a key focus in understanding how these processes unfold.

### 2.1. Neuroplasticity

The idea that experiences lead to brain alterations dates back to the early 1900s when Santiago Ramon y Cajal suggested that learning and experiences may lead to structural changes within the brain [24]. Since then, significant growth in the understanding of neuroplasticity has been made. Neuroplasticity has been categorized in three different ways, based on either specific and expected sensory information or whether sensory information is present or absent [25]. The first type is experience-expectant neuroplasticity, which requires a specific type of sensory information from an expected experience that occurs primarily during the developmental process [25]. To ensure normal development will occur, certain experiences must take place during critical periods, a primary example being language acquisition [26]. Next, experience-independent neuroplasticity occurs without external sensory stimulation and is primarily genetically driven, influenced by spontaneous neural activity [25]. For example, during axon pathfinding, axons project towards potential targets and are guided by chemical cues to form a synapse with a neighboring neuron [27]. The final category is experience-dependent neuroplasticity. Of the three categories, experience-dependent neuroplasticity is most notable as it relates to structural brain changes in response to learning and experience. Experience-expectant and experience-independent neuroplasticity help establish and refine foundational neural structures during development. In contrast, experience-dependent neuroplasticity reflects the ongoing modification of neuronal assemblies that continue throughout life [28].

#### 2.1.1. Experience-Dependent Neuroplasticity

Experience-dependent neuroplasticity can result in both short- and long-term changes. These range from brief alterations in cellular excitability that can last a few milliseconds to hours, to prolonged structural changes that may remain stable over several decades [29,30]. An example of short-term neuroplasticity is working memory. It has been suggested that spiking patterns induced by sensory stimuli lead to changes in synaptic weights within the PFC, leaving a trace of the memory between spikes [31]. The changes in synaptic weights are known as either synaptic depression or facilitation. Facilitation refers to an increase in synaptic strength following repeated stimulation, while depression refers to a decrease, both typically lasting from milliseconds to a few minutes [29]. However, long-term potentiation (LTP) or long-term depression (LTD) will occur if the stimulus is sustained for a prolonged period of time [30]. Repeated stimulation resulting in higher frequency (~100 Hz) will lead to LTP, while LTD can result from low-frequency stimulation (~5 Hz) [29]. Furthermore, repeated high-frequency stimulation will strengthen LTP. For example, saying the name of the person you just met is a form of repeated stimulation. By repeating their name, the LTP between the circuit of neurons associated with that person’s name is strengthened, which in turn would lead to a greater chance you will remember their name in the future. LTP and LTD are generally considered to be forms of functional plasticity, where existing synaptic weights are modified [29].

#### 2.1.2. Neurophysiological Mechanisms of Long-Term Potentiation and Long-Term Depression

The induction of LTP or LTD can be characterized by molecular and morphological changes within neurons [32,33]. Two post-synaptic receptors are related to LTP and LTD: a-amino-3-hydroxy-5-methyl-4-isoxazolepropionate receptors (AMPARs) and N-methyl-D-aspartate receptors (NMDARs) [12,34]. Both types of receptors are responsive to the excitatory neurotransmitter glutamate. Activation of post-synaptic AMPARs allows for the influx of sodium and potassium into the post-synaptic cell, resulting in an excitatory postsynaptic potential (EPSP). NMDARs also permit sodium influx while they are permeable to calcium as well [35]. Interestingly, while the post-synaptic membrane is at its resting potential, magnesium blocks the influx of calcium by attaching to a binding site within the NMDARs [36]. For magnesium to dissociate from NMDARs, the cell must first be depolarized. Once the magnesium is removed from the NMDARs, binding glutamate to the NMDARs initiates the influx of calcium [33]. Both LTP and LTD depend on post-synaptic influx of calcium through NMDARs [33]. The state in which magnesium is dissociated determines whether LTP or LTD is induced.

The complete dissociation of magnesium results in the greatest amount of calcium entering the post-synaptic cell; in turn, a signaling cascade will be initiated, facilitating the migration of AMPARs to the post-synaptic membrane. Increasing the amount of AMPARs improves the efficiency of the synapse, leading to LTP. In contrast, the membrane potential during lower frequency stimulation is moderately depolarized; therefore, magnesium is partially dissociated, allowing for a fraction of the extracellular calcium to enter. The lower intracellular concentration of calcium initiates an alternative signaling cascade that leads to the reduction in post-synaptic AMPARs, resulting in LTD [33]. In the adult brain, the consequential structural changes in LTP and LTD result in enlargement, shrinkage, or elimination of existing synapses [37]. Not only does LTP strengthen existing synapses, but it can also awaken silent synapses [38]. Silent synapses are synapses that contain strictly NMDARs. However, once LTP is induced, these silent synapses begin to insert AMPARs into the post-synaptic membrane, increasing their responsiveness to glutamate [38]. Together, experience-dependent neuroplasticity leads to the functional remodeling of existing local circuits; however, it can also lead to structural changes and neurogenesis, which allows for the creation of new circuits.

It has been demonstrated that experience-dependent neuroplasticity can lead to increases in regional gray matter and cortical thickness [39]. Among the regional alterations are the formation or elimination of dendritic spines, also known as spinogenesis or synaptic pruning, respectively. Additionally, synaptogenesis, sprouting axons, and expanding dendrites are also contributing structural factors in experience-dependent neuroplasticity [40,41]. Notably, in response to learning and practice, these structural changes allow for circuit refinement, efficiency, and stability, ensuring that the most effective and relevant connections are maintained [42]. While these structural changes collectively underpin the brain’s remarkable ability to adapt to novel experiences, another key factor is neurogenesis.

Neurogenesis entails the formation of new neurons. Initially, it was thought that neurogenesis only occurred from the embryonic stage of development to early adulthood. It is now understood, however, that neurogenesis takes place in the adult brain throughout life [43,44]. The neurogenic regions of the adult brain are the subgranular zone of the dentate gyrus in the hippocampus and the subventricular zone lining the lateral ventricles, which supplies new neurons to the olfactory bulb [45]. During novel experiences, neurogenesis within the hippocampus can be modulated by either an increase or a decrease in the number of new neurons following an experience [46]. Stressful events may result in a reduction in neurogenesis, while enriching experiences, even exercise, can lead to an increase in hippocampal neurogenesis [45]. As it relates to experience-dependent neuroplasticity, neurogenesis provides new neurons that can be integrated into existing neural circuits, which may be necessary for acquiring new information and memory recall [44]. These neuroplastic mechanisms provide the biological foundation for adaptation, recovery, and skill refinement. However, their effectiveness may be altered by injury, neurodegenerative disease, and aging, creating conditions in which cognitive–motor function becomes impaired, and targeted intervention may be required.

### 2.2. Challenges to Cognitive–Motor Function in Injury, Neurodegeneration, and Aging

Cognitive–motor function can be disrupted through several biological pathways, including acute injury, progressive disease, and normal age-related decline. Traumatic brain injuries (TBI), neurodegenerative disease (ND), and aging are representative examples of these distinct mechanisms. Together, they illustrate how damage or gradual alterations to neural circuits can impair sensorimotor integration, cognition, motor control, and adaptive capacity, while also highlighting the continued relevance of neuroplasticity for recovery, maintenance, and intervention.

### 2.3. Traumatic Brain Injury

TBI can be penetrating or non-penetrating injuries [47]. Cognitive and motor deficits resulting from a penetrating injury reflect the sustained trauma by a foreign object to a certain region. For example, memory can be affected if the penetrating object causes damage to the hippocampus, while damage to the motor cortex can lead to coordination deficits [48]. In contrast, the mechanical forces experienced during a non-penetrating injury lead to stretching and shearing, causing damage to white matter tracts and cerebro-vasculature [49,50]. The result of these forces can lead to disruptions in metabolic processes and communication between brain regions. Interestingly, the severity of a non-penetrating injury influences the degree and variability of deficits, leading to impairments in balance, fine motor, attention, and impulse control, which are some of the most notable cognitive and motor deficits of a TBI [47,51].

#### 2.3.1. Neurodegenerative Diseases

Cognitive and motor functions are susceptible to injury, ND, and aging. The manifestation of ensuing deficits differs depending on the injury severity and location, disease type, progression, and age. While NDs can have both motor and cognitive deficits, their classification is based on the ND’s most prevalent clinical symptoms. For instance, Parkinson’s disease is considered a movement disorder because its most common consequences include tremors and motor rigidity [52]. Due to common impairments in executive functions and memory, Alzheimer’s disease is known as a cognitive disorder [53]. However, in the advanced stages, individuals with Parkinson’s disease show difficulty with planning and attention alongside their motor impairments, while Alzheimer’s can affect dexterity, fine motor skills, and balance [54,55]. It is important to note that the classification method overlooks coinciding deficits. Recent efforts are being made to adjust the classification approach to increase awareness and ensure each clinical feature is accounted for [56]. The causes of ND-related motor and cognitive deficits are mainly due to the interference of neurodegenerative products on normal cell functioning, resulting in neuronal loss, synaptic dysfunction, and neurotransmitter deficits, leading to reduced resources and disruptions in neural networks [53,54,57]. Importantly, mixed cognitive–motor phenotypes are increasingly recognized as the norm rather than the exception in later disease stages, underscoring the limitations of rigid categorical classifications.

#### 2.3.2. Aging

Cognitive and motor deficits are an inherent part of aging, as over time, reduced brain volume becomes apparent. However, these deficits are due to the gradual loss of neurons, synapses, and synaptic efficiency, unlike TBI and NDs, where a significant amount of neuronal loss occurs within shorter time periods [58]. The cognitive impact of normal aging can be observed in each cognitive domain. Cognitive abilities, however, have been divided into crystallized and fluid abilities to classify the degree of change related to normal aging [59]. Crystallized abilities relate to the capacity to retrieve acquired knowledge and apply it, whereas the ability to absorb external information, cognitively process it, and execute a plan to complete a task requires fluid abilities [60]. Fluid abilities improve into early adulthood and steadily decline to age 80. In contrast, crystallized abilities tend to improve into late adulthood and remain stable until age 80 [59]. However, an interesting finding from Tucker-Drob noted that individuals who demonstrated greater decline in fluid abilities also tended to display reduced improvements or losses in crystallized abilities [60].

Cognitive deficits commonly manifest as memory loss, difficulties in multi-tasking and problem solving, and slower decision-making abilities [59]. Among the cognitive abilities affected by aging, speed of processing is considered a hallmark of cognitive aging, and age-related declines in this domain have been closely associated with motor performance deficits [61,62]. Throughout the aging process, as age-related effects target brain structures, motor functions become more reliant on cognitive functions to compensate. These cognitive functions, however, are also susceptible to aging complications, leading to poorer balance, coordination, movement variability, and gait issues [63]. These changes in motor performance increase the risk of falling progressively in older adulthood and can have detrimental consequences for the health span. Falls represent a common cause of morbidity, reducing the quality of life for individuals who have suffered an injury from falling [64]. Although TBIs, NDs, and age-related decline produce cognitive and motor deficits, each condition retains a varying degree of neuroplasticity, which allows for the recovery, sustainability, and enhancement of these cognitive and motor functions.

#### 2.3.3. Neuroplastic Difference Between TBI, Neurodegenerative Disease, and Aging

The brain’s adaptive ability is maintained throughout a lifetime. Nevertheless, neuroplasticity is known to gradually diminish during the natural aging process [65]. Therefore, the age at which an individual suffers a TBI can influence the extent of recovery. Young adults typically demonstrate a greater capacity to recover from a TBI compared to their younger and older counterparts [48]. Interestingly, unlike aging and TBI, where neuroplastic mechanisms are mainly intact, the pathogenic mechanisms of NDs disrupt neuroplastic mechanisms, reducing the brain’s adaptive capacity and hindering the recovery of cognitive and motor impairments [66]. Because TBI, NDs, and aging can each disrupt cognitive and motor processes while retaining varying degrees of adaptive capacity, interventions that engage these domains may be particularly relevant for rehabilitation and functional maintenance. Accordingly, there is a growing trend toward developing interventions that aim to ameliorate or mitigate cognitive and motor deficits after injury, during ND progression, and in normal aging. Notably, non-invasive strategies such as physical activity and cognitive training have demonstrated the ability to promote neuroplasticity in individuals with TBI, NDs, and aging [66,67,68].

### 2.4. Harnessing Cognitive and Motor Training Interventions for Rehabilitation and Enhancement

#### 2.4.1. Physical Activity

Among non-invasive interventions, physical activity is a notable cost-effective strategy to promote neuroplasticity. Raising the heart rate via aerobic exercise promotes circulation and the transportation of essential resources to the brain, which has acute effects boosting cognitive and motor function momentarily [69]. Moreover, sustained exercise stimulates the release of brain-derived neurotrophic factor (BDNF) and vascular endothelial growth factor (VEGF), which support neuroplastic mechanisms including synaptic plasticity, neurogenesis, and vascular remodeling, ultimately enhancing cognitive and motor functioning [70,71,72]. These effects of physical activity are particularly important for promoting neuroplasticity when affected by an ND and in aging individuals [67,72]. While physical activity is known to enhance several cognitive functions selectively, its capacity to induce the production of prerequisite factors to neuroplasticity indicates that physical activity may play a broader facilitating role for improving cognitive and motor functions [73]. In contrast, cognitive and motor training interventions can directly induce neuroplasticity by engaging targeted neural networks underlying particular domains, such as speed processing, decision-making, and motor coordination [30,74,75]. Therefore, it can be hypothesized that combining both physical activity and cognitive training may lead to better rehabilitation outcomes compared to each intervention alone [73,74]. However, research on physical activity and cognitive training has primarily examined their effects on neuroplasticity and cognitive–motor outcomes separately, with limited attention to the potential benefits of combining these interventions. Cognitive and motor training protocols have undergone extensive exploration, both in terms of their domain-specific targets and the diverse methods through which they are delivered.

#### 2.4.2. Motor Training

Motor learning, a fundamental form of motor training, is a crucial avenue for rehabilitation and performance enhancement. Motor training interventions aim to refine movement execution and coordination through repeated, goal-directed practice. Motor learning is characterized by relatively permanent improvements in motor performance resulting from experience or practice [76]. Traditional motor training protocols often include repetitive task practice, strength and balance exercises, or fine-motor control tasks, all of which promote neuroplastic adaptations within the sensorimotor and cerebellar circuits [77,78]. These interventions are essential for motor recovery following stroke, spinal cord injury, or NDs, where practice-dependent synaptic reorganization supports the functional re-acquisition of lost skills [79,80]. Importantly, growing evidence suggests that motor learning shares common neural mechanisms with cognitive learning, particularly in processes related to attention, error-based learning, and performance monitoring [78,81]. These shared mechanisms suggest that combining motor and cognitive training may lead to synergistic effects, reinforcing the concept of cognitive–motor learning (CML) as a promising framework for rehabilitation and performance enhancement [74,82].

#### 2.4.3. Cognitive Training

Traditional cognitive training approaches refer to non-technological, low-immersion, structured mental practice interventions, such as paper and pencil exercises, which were adapted from tasks originally used for understanding a particular cognitive function, such as using an n-back task to train working memory [83]. Other traditional approaches include using logic puzzles, mnemonic exercises, and card games to train specific cognitive functions [84,85,86]. While traditional methods are still widely used in rehabilitation and research settings [87], many of these cognitive training interventions and their underlying principles have been digitized into computer-based cognitive training (CCT) programs with the advent of advancing technology [88]. Although both traditional and CCT interventions have been known to enhance cognitive functions [89], there are some considerable advantages to using CCT over their traditional counterparts. CCT interventions can be programmed to dynamically adapt the difficulty of the intervention based on the participant’s performance, which, in turn, optimizes the training effects [90]. Furthermore, CCT programs can provide immediate feedback and monitor performance, allowing researchers and clinicians to track the user’s progress in real time [91]. Lastly, many CCT platforms can be accessed by personal computers or mobile devices, making cognitive rehabilitation available at home and reducing dependency on clinic-based sessions and therapist availability [92]. Through these technological advancements and associated advantages, cognitive training has been adopted in both clinical and non-clinical contexts to address a wide range of cognitive deficits and performance goals.

#### 2.4.4. Potential Real-World Implications: Recovery and Enhancement

The potential real-world implications of cognitive and motor training interventions have driven researchers and commercial enterprises to develop potential solutions aimed at restoring, maintaining, and enhancing cognitive and motor function and performance. Consequently, cognitive and motor training has been explored as a non-invasive strategy to ameliorate cognitive deficits across a range of health conditions, including TBI, stroke, multiple sclerosis, Parkinson’s disease, Alzheimer’s disease, and aging [68,93,94,95,96]. Findings from these studies suggest that motor and computerized cognitive training protocols show promise in restoring and maintaining cognitive and motor functions, subsequently improving the quality of life for individuals affected by these conditions [97,98]. Moreover, these benefits extend to a societal level, as improved cognitive and motor health can enable individuals to return to work or remain independent for longer, thereby reducing the burden on healthcare systems and social services [99,100]. Similarly, the notion that enhancing cognitive abilities may contribute to greater professional, athletic, and academic achievement is supported by the established link between higher cognitive functioning and better life outcomes [101,102].

Numerous studies have been conducted to determine whether targeted interventions can enhance cognitive functions. For instance, a study exploring a cognitive training program aimed to enhance working memory and academic performance in children reported that the training improved vocabulary and math performance alongside working memory [103]. Furthermore, cognitive–motor training has been found to be an effective tool for orthopedic surgeons to prevent skill decay during periods of non-performance [104,105].

Recently, cognitive–motor training has also become a substantial focus in the sporting world. There is a significant incentive for sports organizations to promote athletes not only for outperforming their competitors on a physical level but also on a cognitive level [82]. Cognitive and motor functions such as attention, speed of processing, decision-making, and complex coordinated actions are essential for elite performance and have been the primary focus of researchers [82,106]. For attention, researchers found that cognitive training in both lab and field settings led to improvements in the athletes’ visual attention [107], while similar results were also found for speed of processing and decision-making [108,109]. Interestingly, studies that utilized cognitive training protocols targeting both speed of processing and decision-making often report minimal group differences in accuracy [75]. This pattern reflects a speed–accuracy trade-off that may stem from both the participant’s adaptive strategy, where individuals increasingly prioritize speed over precision as training progresses, and from protocol designs that emphasize quick responding over accuracy through feedback or task constraints [75,110]. However, in a competitive sports environment, making decisions faster than the competitors is a substantial advantage, where a split-second decision can mean the difference between scoring and not. While cognitive training interventions and protocols have demonstrated the capacity for improving cognitive functions, the majority of improvements are related to the trained task, which has been a cause of controversy within the field of cognitive training.

### 2.5. Controversies of the Field of Cognitive–Motor Training and Possible Solutions

The central question in the field of cognitive training seeks to determine whether training on a specific cognitive function can be transferred to another domain, and if so, how and to what extent. Gobet and Sala defined cognitive training as an intervention that involves a demanding cognitive task or activity aimed at enhancing general cognitive ability [111]. This concept is also known as far transfer within the cognitive training community. For instance, learning a musical instrument to improve reading comprehension would be an example of far transfer. The counterpart to far transfer is near transfer, which occurs when improvements are seen on tasks that closely resemble the training protocol in structure or cognitive demands [84]. An example of near transfer would be practicing a Sudoku puzzle to enhance performance on other logic puzzles. Although the debate surrounding transfer effects has been most prominent in the cognitive training literature, motor learning research also faces challenges related to transferability, ecological validity, and task specificity. However, these issues have historically received less scrutiny, in part because motor improvements are often assessed in contexts that more closely resemble real-world action.

#### 2.5.1. Debates and Methodological Challenges in Cognitive–Motor Training Research

The field of cognitive training has been marked by ongoing debate regarding the extent to which training-related gains transfer beyond the trained task. A group of 70 scientists from psychology and neuroscience published a letter opposing the claims of the cognitive training industry and its scientific basis, citing examples that overstate the effectiveness of commercial products in transferring improvements to everyday cognitive functioning [112]. In response, 133 scientists, while agreeing that industry claims are exaggerated and misleading, emphasized the importance of exploring the field’s central question by highlighting the growing body of evidence [113]. A central issue underlying these disagreements is the lack of methodological standardization across studies, which complicates comparisons and interpretation of findings [114]. To address this, Green and 47 colleagues argue that disagreements stem from a lack of methodological standards within the field, making it difficult to compare results across studies. As a result, the researchers published a paper offering methodological and best-practice recommendations aimed at improving rigor, transparency, and reproducibility [114].

Despite these earlier efforts, meta-analyses have consistently reported minimal evidence for far transfer, with effect sizes approaching zero, while modest support has been observed for near transfer [111,115]. While some may argue that these results are due to inappropriate comparisons caused by the absence of standardized methods, Gobet and Sala contend that exploring the limits of near transfer should become the new focus of the cognitive training field [111]. Regardless of whether the field will adopt this proposed perspective, establishing a methodological standard for future research is necessary to ensure comparable results between studies and increase the validity of the cognitive training interventions for real-world results. The most notable recommendations emphasize the need to increase statistical power, transparency, and task validity. To achieve this, future research needs to employ adequately powered sample sizes, share data and protocols openly, report null findings, and include multiple measures for each construct to enhance reliability and comparability across studies [111,114,116]. Furthermore, with the recent acceleration in computer-based technology innovation, intervention principles can be integrated into a VR, which may yield better results than previous cognitive training interventions.

#### 2.5.2. Advancing Cognitive–Motor Training Through Virtual Reality

**Figure 2:**
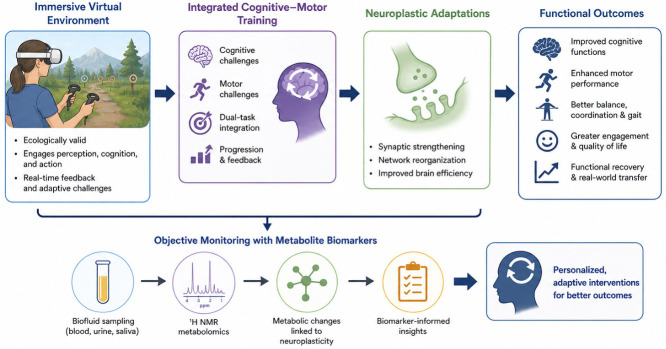
VR-based cognitive–motor training and biomarker-informed adaptation. Immersive VR/VE platforms enable integrated cognitive–motor training that promotes neuroplasticity and improves functional outcomes. Metabolomic profiling (e.g., ^1^H NMR) may provide non-invasive biomarkers of training-induced adaptation, supporting individualized and precision rehabilitation strategies.

Unlike traditional or computer-based cognitive training interventions discussed earlier, the immersive nature of VR offers a novel avenue for CML exploration (Figure 2). VR confers several advantages, including superior ecological validity, a safe and controllable training environment, and the capacity to deliver real-time feedback with adaptive difficulty adjustments. Using head-mounted displays and motion controllers, VR systems enable users to interact naturally with virtual environments (VEs), providing a fully immersive experience through multisensory stimulation [117]. These immersive VEs are designed to facilitate embodied cognition by coupling perception, cognition, and motor output, which are core processes underlying effective CML [118]. The heightened sense of presence associated with VR enhances engagement and attentional focus, further supporting learning and memory consolidation [91]. Furthermore, VEs are highly customizable, allowing researchers to gamify cognitive tasks or replicate real-world scenarios while maintaining the same level of experimental control as laboratory settings. This flexibility enables safe practice of daily living or work-related skills without real-world consequences. For example, individuals recovering from brain injury can use VR to relearn cooking skills, whereas surgeons can refine their techniques between rotations through realistic, interactive simulations [105,119]. Moreover, VR interventions can be designed to deliver immediate performance feedback and dynamically adjust task difficulty, ensuring participants train within an optimal challenge zone [120]. Given these advantages of VR technology and its ecological realism compared to traditional screen-based or paper-and-pencil methods, VR-based cognitive–motor training interventions have demonstrated promising results in promoting transferability of acquired skills and improving functional outcomes in real-world contexts [121,122]. However, VR-based interventions are not without practical limitations. Equipment and software costs, space requirements, and the need for therapist training may restrict access, particularly outside specialized clinical or research settings. Adherence may also depend on usability, patient comfort, and sustained motivation. Therefore, lower-cost standalone systems, simplified interfaces, telerehabilitation, and individualized gamified tasks may help facilitate accessibility and long-term engagement [123]. Further research is needed, however, to determine the extent to which acquired skills transfer to real-world contexts and whether these benefits are maintained over time and across diverse clinical populations.

Despite growing evidence that immersive cognitive–motor training can induce functional improvements, important questions remain regarding the durability, transferability, and biological bases of these effects. VR technology may help address some of these challenges by serving not only as an intervention platform, but also as a tool for assessing and monitoring cognitive–motor performance. During VR training, performance data can be continuously collected, processed, and analyzed to generate digital biomarkers that may assist in detecting early signs of cognitive decline [91]. Nevertheless, behavioral and digital outcomes alone may not fully capture the physiological processes that contribute to individual variability in adaptation. Accordingly, complementary biological approaches, such as ^1^H NMR spectroscopy, may provide additional insight by identifying urinary metabolomic profiles associated with cognitive–motor training. Little is currently known, however, about the specific metabolomic responses elicited by VR-based cognitive–motor training interventions, and further validation is needed before these measures can be considered reliable biomarkers of training-induced adaptation.

### 2.6. NMR-Based Metabolomics and Cognitive–Motor Biomarkers

#### Metabolism and Metabolites

At any given moment, a plethora of continuous chemical reactions are happening within the human body, which are essential to daily function, allowing you, the reader, to see the text and comprehend what has been written. This series of chemical reactions is known as metabolism. Metabolism consists of two contradictory categories of chemical reactions: anabolism, which refers to the synthesis of macromolecules, and catabolism, where macromolecules are broken down into byproducts and building blocks [124]. The process of metabolism is essential for converting the food and nutrients consumed into the materials that are utilized for growth and development [125]. The small-molecule (<1500 Da) substrates, intermediates, and end products of metabolic reactions are known as metabolites, which include peptides, sugars, ketones, steroids, amino acids, and pollutants [126,127,128]. The metabolome, similarly to the genome and proteome, consists of all the metabolites within an organism [129].

Humans possess both an endogenous and an exogenous metabolome, comprising metabolites that are either synthesized naturally within the body or derived directly or indirectly from external sources such as diet, medication, or environmental exposure, respectively. Furthermore, the endogenous metabolome is relatively consistent within humans, whereas the exogenous metabolome can differ substantially [130]. Interestingly, unlike the genome, which is identical across all cell types, each cell type and biofluid has its own specialized metabolome [130]. The metabolome occupies a unique position within the “omics” hierarchy. It serves as an interface between the genome and the environment by capturing both gene-expression products and environmental influences. As a result, metabolomics can provide a sensitive measure of phenotypic expression within the organism [131]. Originating from the metabolome’s phenotyping capacity was an experimental approach coined as metabolomics.

Metabolomics refers to the comprehensive analysis of metabolites within biological fluids and tissues. It can be used to assess changes in metabolite concentrations in response to physiological and external influences, including lifestyle, diet, medication, and environment [132]. To date, the quantification and identification of the entire human metabolome remain incomplete. Nevertheless, substantial gains have been made, with 217,920 metabolites annotated in the most recent version of the Human Metabolome Database (HMDB 5.0) [133]. These gains underscore the progress of metabolomics investigations since the early 2000s, becoming an established means to study environment-gene interactions, drug discovery, and the identification of disease-related biomarkers [128]. Targeted and untargeted methodological approaches are typically implemented during metabolomic studies. A targeted approach utilizing a pre-established list of metabolites related to the pathways of interest is measured and compared; in contrast, an untargeted approach aims to measure the total amount of metabolites within a given sample [134].

In metabolomics, two primary analytical techniques are used to extract metabolite data from a sample of interest: nuclear magnetic resonance (NMR) spectroscopy and mass spectrometry (MS). When paired with liquid chromatography (LC) or gas chromatography (GC), MS enables the detection and quantification of a broad range of molecular species [135]. Both techniques have their advantages and disadvantages as they relate to metabolomics. NMR is a non-destructive technique that requires minimal sample preparation with high reproducibility, allowing for the saving and re-analysis of a sample for an extended period of time [136]. In contrast, MS is a destructive technique that requires more extensive sample preparation and resources. Its results are less reproducible than those obtained by NMR. However, MS is considerably more sensitive and can detect upwards of a thousand different metabolites [137]. While MS offers higher sensitivity, NMR has the ability to detect and quantify approximately 200 metabolites in a single measurement [136,138]. Although NMR offers lower sensitivity compared to mass spectrometry, this characteristic simplifies metabolomic analysis by focusing on known key metabolites.

NMR-based metabolomics produces substantial datasets; in urine samples, the 150–200 detectable metabolites can yield over a thousand overlapping spectral peaks and hundreds of thousands of data points per spectrum [139,140,141]. Before multivariate statistics can be performed, several pre-processing steps are used to simplify the spectral data, including peak alignment and binning [141]. From this, significant bins can be identified and compared between two samples collected at different times and/or compared to controls [142]. These compounds are then compared to a metabolite database to identify the metabolites of interest. Based on this procedure, NMR has become a crucial tool for metabolomics for identifying potential biomarkers related to disease, sports-related concussion, traumatic brain injury, and cognition by utilizing biofluids and tissues, namely blood, neural tissue, and urine [143,144,145,146,147]. The identified metabolites also provide key insights into the biochemical mechanisms that are affected by disease, injury, external stimuli, and environmental conditions.

Together, the integration of immersive VR and VEs within CML frameworks may provide a systems-level approach to rehabilitation by engaging perception, cognition, and action within ecologically valid contexts. While these interventions show promise for improving functional outcomes, current evidence remains limited by variability in study design, intervention duration, outcome measurements, and evidence for transfer beyond the trained task. In this context, ^1^H NMR-based metabolomics may offer a complementary approach for characterizing systemic biochemical signatures associated with training. However, urinary metabolomic profiles should not be interpreted as a direct measure of neuroplasticity. Rather, they may reflect broader physiological processes related to training-induced adaptations. Further longitudinal studies with standardized protocols and appropriate control and experimental groups are required before metabolomic profiles can be used to identify training responders or guide individualized rehabilitation strategies. With further validation, integrating metabolomic profiling with VR-based cognitive–motor interventions may help characterize biological responses to training, refine intervention strategies, and eventually contribute to precision rehabilitation paradigms that are both behaviorally effective and biologically informed.

## 3. Conclusions and Future Outlook

Human interaction with the environment depends on the continuous integration of sensory input, cognitive processing, and motor output within dynamic sensorimotor loops. Across the lifespan, these interactions are shaped by learning, memory, and neuroplastic mechanisms that allow individuals to adapt to changing environmental demands, recover from injury or disease, and optimize performance. This narrative state-of-the-art review highlights how these processes are instantiated across distributed neural circuits and how experience-dependent plasticity supports both functional recovery and enhancement in health and disease. Furthermore, it considers how cognitive–motor training may support recovery and performance optimization, and how ^1^H NMR-based metabolomics may provide complementary insight into training-related physiological changes.

A growing body of evidence indicates that non-invasive interventions, including physical activity, motor training, cognitive training, and increasingly immersive technologies such as VR, can harness neuroplastic mechanisms to improve cognitive and motor outcomes. However, variability in training efficacy, transferability, and real-world relevance remains a central challenge. Addressing these limitations will require more rigorous methodological standards, ecologically valid task designs, and integrative frameworks that align neural mechanisms with functional outcomes.

Moving forward, combining immersive cognitive–motor training with objective biological measures represents a promising avenue for advancing the field. Technologies such as VR offer the ability to engage perception, cognition, and action simultaneously within controlled yet realistic environments, while metabolomics, particularly NMR-based approaches, may provide sensitive, non-invasive biomarkers of neuroplastic adaptation. Integrating behavioral, neurophysiological, and metabolic data has the potential to improve intervention personalization, monitor training responsiveness, and identify early markers of recovery or decline. Together, these converging approaches support a shift toward precision cognitive–motor interventions grounded in systems neuroscience and biological phenotyping. Such integration may ultimately enhance rehabilitation strategies, promote resilience across the lifespan, and improve functional independence in both clinical and non-clinical populations.

## Data Availability

No new data were created or analyzed in this study. Data sharing is not applicable to this article.
